# Development and Validation of a Deep Learning Method to Predict Cerebral Palsy From Spontaneous Movements in Infants at High Risk

**DOI:** 10.1001/jamanetworkopen.2022.21325

**Published:** 2022-07-11

**Authors:** Daniel Groos, Lars Adde, Sindre Aubert, Lynn Boswell, Raye-Ann de Regnier, Toril Fjørtoft, Deborah Gaebler-Spira, Andreas Haukeland, Marianne Loennecken, Michael Msall, Unn Inger Möinichen, Aurelie Pascal, Colleen Peyton, Heri Ramampiaro, Michael D. Schreiber, Inger Elisabeth Silberg, Nils Thomas Songstad, Niranjan Thomas, Christine Van den Broeck, Gunn Kristin Øberg, Espen A.F. Ihlen, Ragnhild Støen

**Affiliations:** 1 Department of Neuromedicine and Movement Science, Norwegian University of Science and Technology, Trondheim, Norway; 2 Department of Clinical and Molecular Medicine, Norwegian University of Science and Technology, Trondheim, Norway; 3 Clinic of Clinical Services, St Olavs Hospital, Trondheim University Hospital, Trondheim, Norway; 4 Department of Computer Science, Norwegian University of Science and Technology, Trondheim, Norway; 5 Ann and Robert H. Lurie Children’s Hospital of Chicago, Chicago, Illinois; 6 Northwestern University Feinberg School of Medicine, Chicago, Illinois; 7 Shirley Ryan AbilityLab, Chicago, Illinois; 8 Division of Paediatric and Adolescent Medicine, Oslo University Hospital, Oslo, Norway; 9 Section of Developmental and Behavioral Pediatrics, University of Chicago, Comer Children’s Hospital, Chicago, Illinois; 10 Kennedy Research Center on Neurodevelopmental Disabilities, University of Chicago, Comer Children’s Hospital, Chicago, Illinois; 11 Department of Rehabilitation Sciences and Physiotherapy, Ghent University, Ghent, Belgium; 12 Department of Pediatrics, University of Chicago, Comer Children’s Hospital, Chicago, Illinois; 13 Department of Pediatrics and Adolescent Medicine, University Hospital of North Norway, Tromsø, Norway; 14 Department of Neonatology, Christian Medical College Vellore, Vellore, Tamil Nadu, India; 15 Department of Health and Care Sciences, Faculty of Health Sciences, The Arctic University of Norway, Tromsø, Norway; 16 Department of Neonatology, St Olavs Hospital, Trondheim University Hospital, Trondheim, Norway

## Abstract

**Importance:**

Early identification of cerebral palsy (CP) is important for early intervention, yet expert-based assessments do not permit widespread use, and conventional machine learning alternatives lack validity.

**Objective:**

To develop and assess the external validity of a novel deep learning–based method to predict CP based on videos of infants’ spontaneous movements at 9 to 18 weeks’ corrected age.

**Design, Setting, and Participants:**

This prognostic study of a deep learning–based method to predict CP at a corrected age of 12 to 89 months involved 557 infants with a high risk of perinatal brain injury who were enrolled in previous studies conducted at 13 hospitals in Belgium, India, Norway, and the US between September 10, 2001, and October 25, 2018. Analysis was performed between February 11, 2020, and September 23, 2021. Included infants had available video recorded during the fidgety movement period from 9 to 18 weeks’ corrected age, available classifications of fidgety movements ascertained by the general movement assessment (GMA) tool, and available data on CP status at 12 months’ corrected age or older. A total of 418 infants (75.0%) were randomly assigned to the model development (training and internal validation) sample, and 139 (25.0%) were randomly assigned to the external validation sample (1 test set).

**Exposure:**

Video recording of spontaneous movements.

**Main Outcomes and Measures:**

The primary outcome was prediction of CP. Deep learning–based prediction of CP was performed automatically from a single video. Secondary outcomes included prediction of associated functional level and CP subtype. Sensitivity, specificity, positive and negative predictive values, and accuracy were assessed.

**Results:**

Among 557 infants (310 [55.7%] male), the median (IQR) corrected age was 12 (11-13) weeks at assessment, and 84 infants (15.1%) were diagnosed with CP at a mean (SD) age of 3.4 (1.7) years. Data on race and ethnicity were not reported because previous studies (from which the infant samples were derived) used different study protocols with inconsistent collection of these data. On external validation, the deep learning–based CP prediction method had sensitivity of 71.4% (95% CI, 47.8%-88.7%), specificity of 94.1% (95% CI, 88.2%-97.6%), positive predictive value of 68.2% (95% CI, 45.1%-86.1%), and negative predictive value of 94.9% (95% CI, 89.2%-98.1%). In comparison, the GMA tool had sensitivity of 70.0% (95% CI, 45.7%-88.1%), specificity of 88.7% (95% CI, 81.5%-93.8%), positive predictive value of 51.9% (95% CI, 32.0%-71.3%), and negative predictive value of 94.4% (95% CI, 88.3%-97.9%). The deep learning method achieved higher accuracy than the conventional machine learning method (90.6% [95% CI, 84.5%-94.9%] vs 72.7% [95% CI, 64.5%-79.9%]; *P* < .001), but no significant improvement in accuracy was observed compared with the GMA tool (85.9%; 95% CI, 78.9%-91.3%; *P* = .11). The deep learning prediction model had higher sensitivity among infants with nonambulatory CP (100%; 95% CI, 63.1%-100%) vs ambulatory CP (58.3%; 95% CI, 27.7%-84.8%; *P* = .02) and spastic bilateral CP (92.3%; 95% CI, 64.0%-99.8%) vs spastic unilateral CP (42.9%; 95% CI, 9.9%-81.6%; *P* < .001).

**Conclusions and Relevance:**

In this prognostic study, a deep learning–based method for predicting CP at 9 to 18 weeks’ corrected age had predictive accuracy on external validation, which suggests possible avenues for using deep learning–based software to provide objective early detection of CP in clinical settings.

## Key Points

**Question:**

What is the external validity of a deep learning–based method to predict cerebral palsy (CP) based on infants’ spontaneous movements at 9 to 18 weeks’ corrected age?

**Findings:**

In this prognostic study of 557 infants with a high risk of perinatal brain injury, a deep learning–based method for early prediction of CP had sensitivity of 71%, specificity of 94%, positive predictive value of 68%, and negative predictive value of 95%. Prognosis of CP based on the deep learning–based method was associated with later functional level and CP subtype in children with CP.

**Meaning:**

This study’s findings suggest that deep learning–based assessments could support early detection of CP in infants at high risk.

## Teaser

This prognostic study describes the development of a deep learning–based method to predict cerebral palsy based on videos of infants’ spontaneous movements at 9 to 18 weeks’ corrected age and assesses the external validity of the deep learning prediction model.

## Introduction

Cerebral palsy (CP) is the most common physical disability in children, producing functional limitation and co-occurring impairments^1^ (eg, pain, musculoskeletal deformities, seizures, and communication and sleep disorders) because of injury to the developing brain.^2^ Cerebral palsy is typically diagnosed between ages 12 and 24 months, and milder forms of CP may be diagnosed even later in childhood.^3,4^ Early identification of infants with a high risk of CP is important to provide targeted follow-up and interventions during infancy when neuroplasticity is high,^5,6^ improve access to community services to minimize complications,^7^ and reassure parents of infants at high risk if their children are unlikely to develop CP.^8^

Prechtl et al^9,10,11^ introduced the general movement assessment (GMA) tool 25 years ago as a method to predict CP. From birth until 2 months’ corrected age, general movements (ie, spontaneous movements involving the whole body) have a writhing character and later occur as fidgety movements.^12^ The GMA is recommended as the most accurate clinical test for CP prognosis among infants younger than 5 months^4,13^ based on the absence of the fidgety type of general movements.^14,15^ The GMA is based on clinical experts’ observation of infants’ general movements in video recordings. The method requires training,^16^ and rater experience may alter GMA reliability.^17^ These factors hamper widespread clinical use.^18^

With advancements in the field of artificial intelligence, machine learning techniques have been developed as objective low-cost alternatives to the GMA.^18,19,20,21,22^ Former machine learning techniques for tracking and classification of infants’ spontaneous movements generally aimed to predict CP by proposing restricted sets of manually selected movement features used in combination with conventional statistical methods (eg, logistic regression analysis and support vector machines).^23,24,25,26,27^ A recent study^28^ found that the predictive values of these conventional machine learning–based CP prediction models were similar to the predictive values of the GMA. Despite this progress, there are fundamental challenges yet to be addressed. The restricted set of manually selected movement features has an unknown association with the observational GMA tool, which calls into question the construct validity of conventional machine learning techniques. External validation is consequently lacking because of small samples and short follow-up duration.^18,21^ As a result, validation is performed using less conservative methods (including leave-one-out cross-validation) and the absence of fidgety movements as a surrogate predictor for CP.^18,29^

A new field within machine learning, called deep learning, has enabled automatic detection of discriminative movement features through representation learning.^30^ This process involves dynamically selecting features relevant to the task at hand without any human expert involvement. The accuracy of deep learning improves with increasing amounts of data (eg, videos), and deep learning has the capacity to detect features representing intricate associations in the data, such as complex full-body general movements.

Our primary objective was to develop a deep learning–based early prediction model of CP based on infants’ spontaneous movements during the fidgety movement period from 9 to 18 weeks’ corrected age and to perform external validation using a multicenter sample of infants with a high risk of perinatal brain injury. Our secondary objective was to compare the predictive accuracy of the deep learning–based prediction method with the accuracy of the clinically recommended GMA tool and the conventional machine learning method and to evaluate the ability of the deep learning method to predict functional level and CP subtype.

## Methods

### Participants

This prognostic study of patients at 13 hospitals was approved by the regional Committee for Medical and Health Research Ethics in Norway and local institutional review boards in Belgium, India, and the US. Written informed consent was obtained from parents before study inclusion (including written parental consent for publication of an infant image). This study followed the Transparent Reporting of a Multivariable Prediction Model for Individual Prognosis or Diagnosis (TRIPOD) reporting guideline.

The sample comprised 557 infants with a high risk of perinatal brain injury who were prospectively enrolled in previous studies^27,31,32,33^ of CP risk prediction between September 10, 2001, and October 25, 2018. Statistical analysis was performed between February 11, 2020, and September 23, 2021. A description of these previous studies is available in eAppendix 1 in the Supplement. All studies included infants with an increased risk of abnormal neurodevelopment, which was identified before discharge from the neonatal intensive care unit (eAppendix 2 and eTable 1 in the Supplement). Infants were included based on the following criteria: (1) available video following the standards of the Prechtl GMA tool^34^ recorded during the fidgety movement period from 9 to 18 weeks’ corrected age, (2) available GMA classifications of fidgety movements, and (3) available data on CP status at 12 months’ corrected age or older. Two infants with videos recorded at 7 weeks’ and 8 weeks’ corrected age were included. Both were correctly classified by the GMA; 1 infant had intermittent fidgety movements and did not develop CP, and 1 infant had absent fidgety movements and was diagnosed with CP. Data on infants excluded because of missing video recordings, GMA classification, or CP status are reported elsewhere.^27,31,32,33^ The sample size was determined by the number of infants from the previous studies^27,31,32,33^ who had available data.

### Videos and Classification of General Movements

Infants were recorded in the supine position during active wakefulness for a median of 5 minutes (range, 1-9 minutes) following GMA standards.^34^ A conventional video camera (Sanyo VPC-HD2000 Xacti dual camera [Funai Corporation] or Sony Handycam DCR-PC100E [Sony Electronics Inc]) at a median recording rate of 30 frames per second (range, 24-60 frames per second) and a median video resolution of 720 × 1280 pixels (range, 576 × 720 to 1080 × 1920 pixels) was used in a standardized setup comprising a mattress and a stationary overhead camera. If more than 1 recording was available, the recording made between 12 weeks’ and 13 weeks’ corrected age was used.

Two certified observers (L.A. and T.F.) who were blinded to the medical history of the infants performed classification of fidgety movements for all infants. Fidgety movements were classified as normal (sporadically, intermittently, or continuously present) or abnormal (absent). Classification of sporadic fidgety movements as normal was based on a previous study that found a low risk of CP among infants with sporadic fidgety movements.^31^ Infants classified with exaggerated fidgety movements that were excessive in amplitude and speed were excluded a priori from the analysis because of unpredictable outcomes among infants in this category. In cases of disagreement between observers, videos were reassessed by the same 2 observers, and consensus was reached.

### Cerebral Palsy Status, Subtype, and Functional Level

The primary outcome of CP was diagnosed by a pediatrician who was unaware of GMA classifications and followed the CP decision tree of the Surveillance of Cerebral Palsy in Europe.^35^ This diagnosis included classification of CP subtypes into spastic unilateral, spastic bilateral, dyskinetic, and ataxic. Follow-up times differed between studies, ranging from ages 18 months to 5 years.^27,31,32,33^ The Gross Motor Function Classification System (GMFCS; levels I-V, with level I indicating the ability to walk without limitations; level II, the ability to walk with limitations; level III, the ability to walk using a handheld mobility device; level IV, the ability for self-mobility with limitations [may need to use powered mobility]; and V, the need to be transported in a manual wheelchair)^3^ was used to classify functional levels into ambulatory CP (levels I, II, and III) and nonambulatory CP (levels IV and V).

### Method Development and External Validation

**Figure 1.**
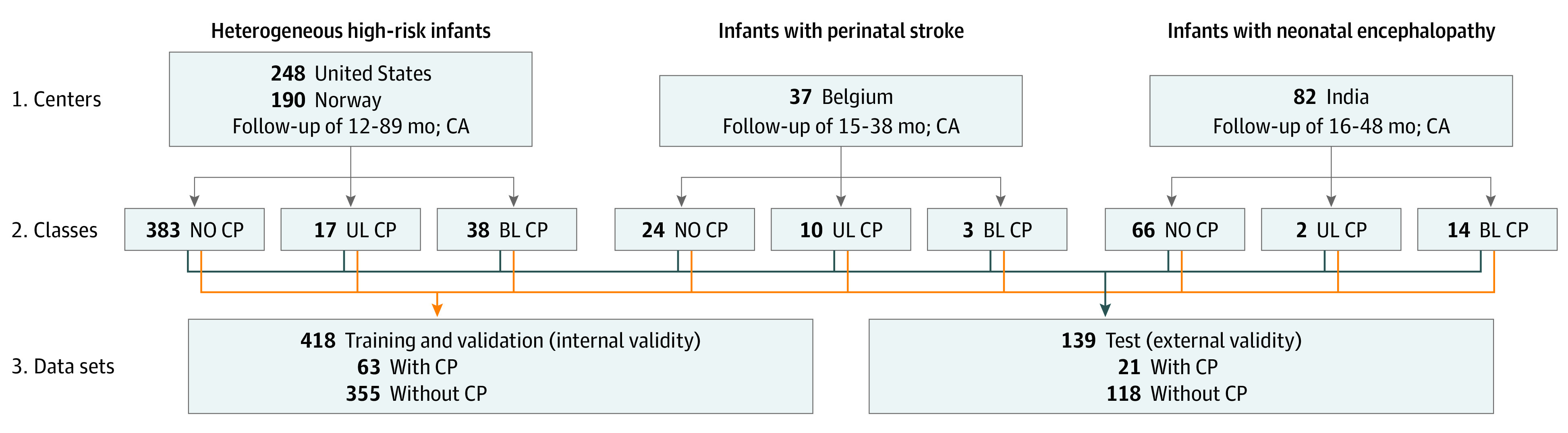
Data Sets for Development and External Validation Infants diagnosed with cerebral palsy (CP) for whom subtype was not available were classified as having spastic unilateral CP (UL CP) if they had a Gross Motor Function Classification System level of I or II and classified as having spastic bilateral CP (BL CP) if they had a Gross Motor Function Classification System level of III, IV, or V. Infants with dyskinetic CP and ataxic CP were classified as having BL CP. A total of 75.0% of infants of each class (orange path in step 3) were randomly assigned to the method development (training and internal validation) sample, and the remaining 25.0% were randomly assigned (blue path in step 3) to the external validation sample. CA indicates corrected age.

To achieve representative samples for method development (ie, training and internal validation) and external validation, all infants at high risk were stratified into classes based on the study in which they were originally enrolled,^27,31,32,33^ the country of the center in which the study was conducted (Belgium, India, Norway, or the US) (step 1 in Figure 1), and their CP subtype (spastic bilateral CP, spastic unilateral CP, or no CP) (step 2 in Figure 1). Data on race and ethnicity were not reported because the previous studies (from which the infant samples were derived) used different study protocols with inconsistent collection of these data.

A total of 75.0% of infants of each class (orange path in step 3 of Figure 1) were randomly assigned to the method development (training and internal validation) sample, and the remaining 25.0% were randomly assigned (blue path in step 3 of Figure 1) to the external validation sample (1 test set). Infants assigned to the method development sample were further divided into 7 internal validation samples (ie, folds), each comprising 9 infants with CP and 50 or 51 infants without CP. This additional division enabled 7-fold cross-validation for evaluating internal validity. The internal validation samples were constructed using a similar procedure for stratification based on study center and CP subtype (as performed with the external validation test set shown in Figure 1).

### Deep Learning Method

**Figure 2.**
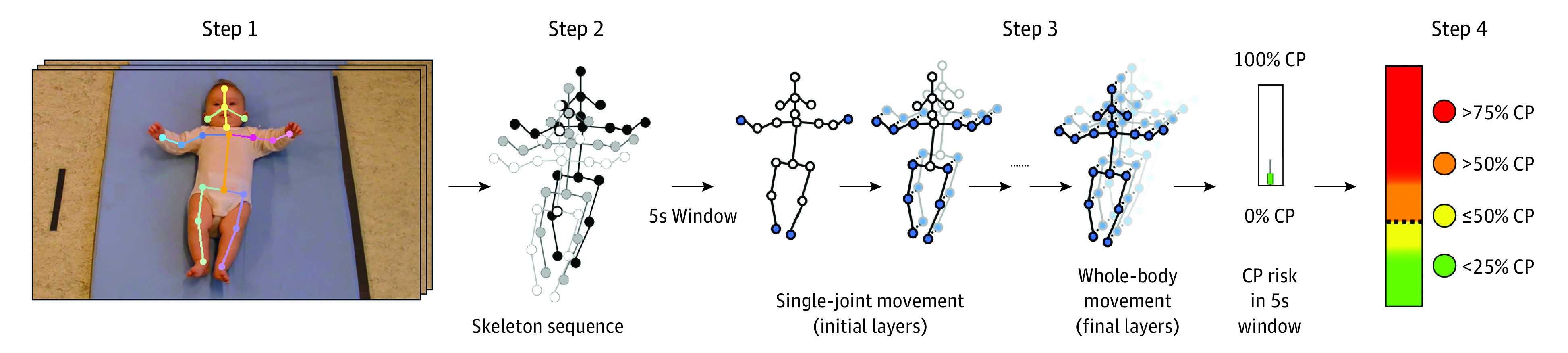
Steps Involved in Deep Learning–Based Method for Cerebral Palsy Prediction In the deep learning–based method, a video-based infant motion tracker (step 1) constructs a skeleton sequence of 5-second (5s) windows (step 2), in which a deep learning–based prediction model estimates cerebral palsy (CP) risk in each 5-second window by detecting single-joint movements over a few time steps in the initial model layers and whole-body movements over many time steps in the later model layers (step 3). Next, CP risk of the total video is aggregated to classify an infant as having CP or no CP (step 4) based on the decision threshold (dashed line) and yield uncertainty of classification (color coding, with red representing certain classification of CP, orange representing uncertain classification of CP, yellow representing uncertain classification of no CP, and green representing certain classification of no CP). Written parental consent was obtained for publication of the infant image in step 1.

The overall concept of the deep learning method for CP prediction is presented in Figure 2. The method comprised 4 steps: (1) motion tracking, (2) creation of a skeleton sequence, (3) development of a deep learning–based prediction model, and (4) prediction of CP.

#### Motion Tracking

The raw video was processed by a motion tracker^36^ that localized horizontal and vertical coordinates of 19 important body points (forehead, nose, ears, upper neck, shoulders, elbows, wrists, upper chest, right pelvis, left pelvis, midpelvis, knees, and ankles) (step 1 in Figure 2), creating an infant skeleton (ie, a full pose of the infant in the form of a digital skeleton model). The motion tracker had previously been trained and validated on infant videos according to GMA standards following in-motion poses as defined by Groos et al^37^; further technical details of the motion tracker are described in the original articles by Groos et al.^36,37^

#### Skeleton Sequence

The infant skeletons of all video frames composed a spatiotemporal skeleton sequence (step 2 in Figure 2) representing infant movements in the video. The skeleton sequence was divided into 5-second windows, which were processed by the deep learning–based prediction model to estimate CP risk in that particular window.

#### Deep Learning–Based CP Prediction Model

To automatically detect movement features associated with CP, a novel deep learning procedure was developed. A deep learning model consists of multiple layers (step 3 in Figure 2). The initial layers detect features of movements of a single limb or joint, whereas subsequent layers detect features of whole-body movements. To prevent manual selection bias, the optimal model architecture was set by an automatic search on the training and internal validation data. The first 10 automatically selected models were defined as artificial experts and retrained on the 7 internal validation samples (internal validation results are provided in eTable 2 in the Supplement). Each of the resulting 70 artificial expert instances used the biomechanical properties (position, velocity, and body segment length) in 5-second windows to detect whole-body movement features that distinguished infants with CP from infants without CP. Details on the automatic search procedure and configurations of selected deep learning models are available in eAppendix 3, eTable 3, eTable 4, and eFigure 1 in the Supplement.

With regard to the group of artificial experts and uncertainty of decisions, based on the obtained movement features in each of the 70 artificial expert instances, CP risk was estimated on a continuous scale from low (0%) to high (100%). The median value of the 70 individual artificial expert predictions was used as CP risk in the 5-second window, with uncertainty of CP risk color coded based on the level of agreement across the 70 predictions. Green (with 0-17 agreements [<25.0%] predicting CP) and yellow (with 18-35 agreements [≤50.0%] predicting CP) represented certain and uncertain predictions of no CP, respectively. Orange (with 36-52 agreements [>50.0%] predicting CP) and red (with 53-70 agreements [>75.0%] predicting CP) represented uncertain and certain predictions of CP, respectively.

#### Prediction of CP

The final score for CP risk in a total video was estimated as the median CP risk across all 5-second windows of the skeleton sequence (step 4 in Figure 2). This score was used to classify an infant as having CP or no CP based on a fixed decision threshold (different thresholds are shown in eAppendix 4, eFigure 2, and eTable 5 in the Supplement). A classification of CP was considered certain (red) if more than 75.0% of the artificial expert predictions were classified as CP and uncertain (orange) if more than 50.0% were classified as CP. A classification of no CP was considered uncertain (yellow) if 50.0% or fewer of the artificial expert predictions were classified as CP and certain (green) if fewer than 25.0% were classified as CP (step 4 in Figure 2).

### Conventional Machine Learning Method

To enable objective comparison between the deep learning method and the conventional machine learning method previously described,^28^ retraining of the conventional machine learning method was performed on skeleton sequences of 19 important body points in the method development data set. Additional details about the conventional machine learning method have been published previously by Ihlen et al.^28^

### Statistical Analysis

The sensitivity of the methods used for external validation was fixed a priori based on the sensitivity level of the GMA tool to ensure objective comparisons. The Clopper-Pearson method was used to calculate 95% CIs for sensitivity, specificity, positive and negative predictive value, and accuracy, which were computed using the conf package in R software, version 4.0 (R Foundation for Statistical Computing). The difference in CP risk between infants with ambulatory CP (GMFCS level I, II, or III) and nonambulatory CP (GMFCS level IV or V) was assessed using a 2-sided Wilcoxon rank sum test and computed using algorithms from the SciPy library in Python, version 3.6 (Python Software Foundation). A Wilcoxon rank sum test was also used to assess the difference in CP risk among infants with spastic unilateral CP vs spastic bilateral CP. The significance threshold was 2-tailed *P* < .05.

## Results

Among 557 infants at high risk, 310 (55.7%) were male, and 247 (44.3%) were female; the median (IQR) corrected age at assessment was 12 (11-13) weeks, and 84 infants (15.1%) had a diagnosis of CP at a mean (SD) age of 3.4 (1.7) years (eTable 1 in the Supplement). The median (IQR) corrected age at which CP status was evaluated was 38 (23-46) months. A total of 418 infants (75.0%) were randomly assigned to the model development sample, and 139 (25.0%) were randomly assigned to the external validation sample.

Predictive accuracies of the deep learning method, the GMA tool, and the conventional machine learning method are presented in the Table. On external validation, the deep learning–based CP prediction method had sensitivity of 71.4% (95% CI, 47.8%-88.7%), specificity of 94.1% (95% CI, 88.2%-97.6%), positive predictive value of 68.2% (95% CI, 45.1%-86.1%), and negative predictive value of 94.9% (95% CI, 89.2%-98.1%). In comparison, the GMA tool had sensitivity of 70.0% (95% CI, 45.7%-88.1%), specificity of 88.7% (95% CI, 81.5%-93.8%), positive predictive value of 51.9% (95% CI, 32.0%-71.3%), and negative predictive value of 94.4% (95% CI, 88.3%-97.9%). The deep learning method achieved higher accuracy than the conventional machine learning method (90.6% [95% CI, 84.5%-94.9%] vs 72.7% [95% CI, 64.5%-79.9%]; *P* < .001), but no significant improvement in accuracy was observed compared with the GMA tool (85.9%; 95% CI, 78.9%-91.3%; *P* = .11).

**Table.** Predictive Values on External Validation Given a Fixed Sensitivity of 70.0%^a^

Method	Result, No.				Validation measure, % (95% CI)				
	True positive	False positive	True negative	False negative	Sensitivity	Specificity	PPV	NPV	Accuracy
Deep learning	15	7	111	6	71.4 (47.8-88.7)	94.1 (88.2-97.6)	68.2 (45.1-86.1)	94.9 (89.2-98.1)	90.6 (84.5-94.9)
GMA	14	13	102	6	70.0 (45.7-88.1)	88.7 (81.5-93.8)	51.9 (32.0-71.3)	94.4 (88.3-97.9)	85.9 (78.9-91.3)
Conventional machine learning	15	32	86	6	71.4 (47.8-88.7)	72.9 (63.9-80.7)	31.9 (19.1-47.1)	93.5 (86.3-97.6)	72.7 (64.5-79.9)

Abbreviations: GMA, general movement assessment tool; NPV, negative predictive value; PPV, positive predictive value.

^a^ The external validation sample included 4 infants (1 with cerebral palsy and 3 without cerebral palsy) with exaggerated fidgety movements (excluded by the GMA), yielding 3 true-negative results and 1 false-negative result, both with deep learning–based and conventional machine learning–based predictions of cerebral palsy. Sensitivity was fixed based on the sensitivity level of the GMA tool.

**Figure 3.**
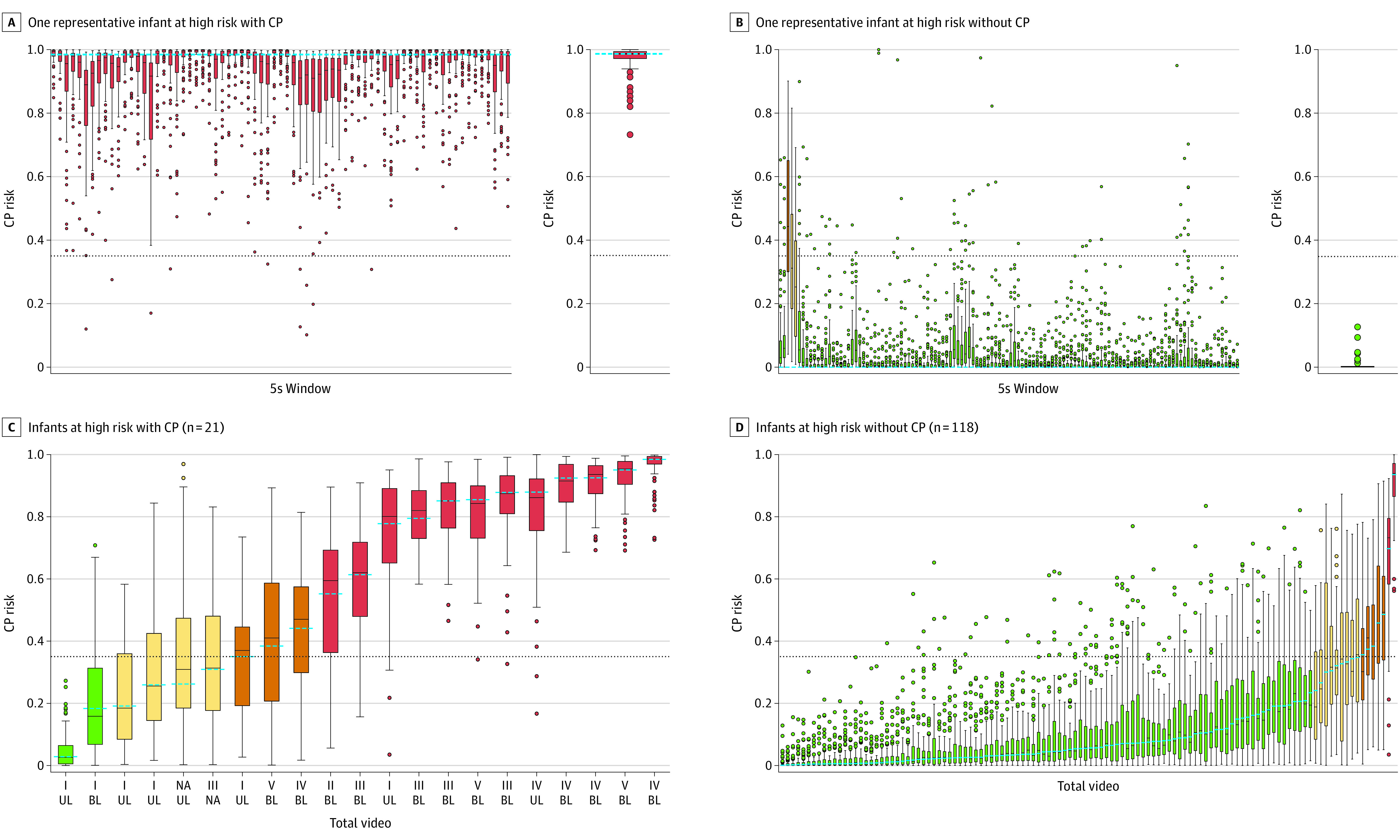
Risk Predictions for Infants in the External Validation Sample A total of 139 infants were included. A and B, cerebral palsy (CP) risk in 5-second (5s) windows is shown on the left, and aggregated CP risk across the total video is shown on the right. The dashed horizontal line represents aggregated CP risk. Both of the representative infants at high risk were classified correctly with high classification certainty. C and D, Distribution of individual CP risk and box plots of classification uncertainties of the 70 artificial expert predictions among infants at high risk with and without CP. The dots indicate outlier points. In C, the x-axis displays the Gross Motor Function Classification System level (with levels I, II, and III indicating ambulatory CP and levels IV and V indicating nonambulatory CP) and the CP subtype (spastic unilateral [UL] or spastic bilateral [BL]) at the time of diagnosis. In the box plots, the dashed blue horizontal lines represent aggregated CP risk, the solid black horizontal lines represent median CP risk across artificial experts, lower and upper edges represent IQR, and whiskers represent range (or 1.5 times the IQR). The dashed horizontal line running across each graph represents the decision threshold. Red represents certain classification of CP, orange represents uncertain classification of CP, yellow represents uncertain classification of no CP, and green represents certain classification of no CP. NA indicates not available.

The external validation sample comprised 139 infants; of those, 21 infants (15.1%) were diagnosed with CP. Among those diagnosed with CP, 12 infants (57.1%) were correctly classified as having certain CP, and 2 infants (9.5%) were incorrectly classified as having certain no CP (red and green box plots in Figure 3C). Of 118 infants without CP, 104 (88.1%) were correctly classified as having certain no CP, and 2 (1.7%) were incorrectly classified as having certain CP (green and red box plots in Figure 3D). Cerebral palsy risk across 5-second windows for 1 representative infant with CP and 1 representative infant without CP, both classified correctly with high certainty, are shown in Figure 3A and B.

**Figure 4.**
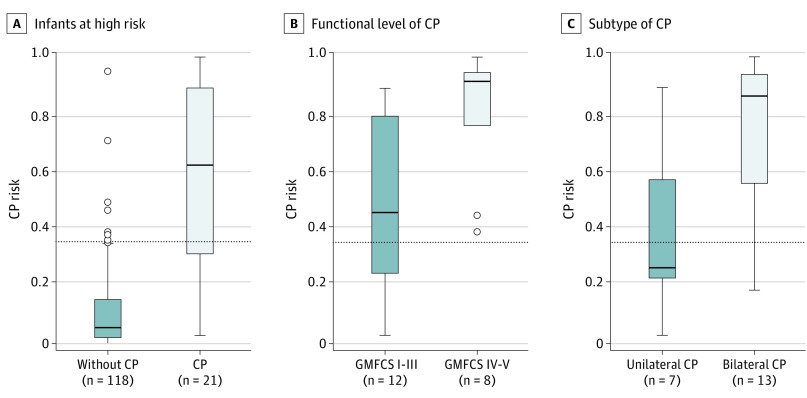
Cerebral Palsy (CP) Risk Among Infants in the External Validation Sample With Different Outcomes Distribution of CP risk across 139 infants. In box plots, the solid black horizontal lines represent median aggregated CP risk, lower and upper edges represent IQR, and whiskers represent range (or 1.5 times the IQR). The dots indicate outlier points. The dashed black horizontal lines represent the decision threshold. Gross Motor Function Classification System levels I through III indicate ambulatory CP, and levels IV and V indicate nonambulatory CP.

The deep learning–based CP prediction method had higher sensitivity (ie, a greater percentage of infants higher than the decision threshold) among infants with nonambulatory CP (100%; 95% CI, 63.1%-100%) vs ambulatory CP (58.3%; 95% CI, 27.7%-84.8%; *P* = .02) and among infants with spastic bilateral CP (92.3%; 95% CI, 64.0%-99.8%) vs spastic unilateral CP (42.9%; 95% CI, 9.9%-81.6%; *P* < .001) (Figure 3C). A significantly higher estimated CP risk was observed among infants with nonambulatory motor function (median [IQR], 0.90 [0.75-0.93]) vs ambulatory motor function (median [IQR], 0.45 [0.24-0.78]; *P* = .007) and among infants with spastic bilateral CP (median [IQR],0.85 [0.55-0.92]) vs spastic unilateral CP (median [IQR], 0.26 [0.23-0.56]; *P* = .03) (Figure 4).

## Discussion

In this prognostic study, a fully automated deep learning method for early CP prediction had high predictive accuracy in an external sample of infants from different countries with heterogenous perinatal risk factors and variation in video quality. Furthermore, the deep learning method differentiated between infants who developed ambulatory vs nonambulatory CP as well as spastic unilateral vs spastic bilateral CP. This novel method may support decisions in early pediatric care by initiating targeted interventions to improve function, prevent complications, and individualize follow-up. This prediction method is a substantial improvement compared with previously published conventional machine learning–based CP prediction methods.^28^

The improved predictive accuracy of the deep learning method compared with the conventional machine learning method used in a previous study^28^ may have occurred for several reasons. First, the deep learning method has the capacity to identify intricate associations in the data because it processes data in several layers. This ability suggests that deep learning could handle the high complexity and variation observed in infants’ spontaneous movements. Second, manual selection of movement features, which is required when using conventional machine learning methods,^25,26^ is eliminated by the automatic feature detection of deep learning methods. Although previous studies have also used deep learning methods for classification of infant motor function,^38,39,40,41^ these studies have been limited by small samples and lack of external validation and have used surrogate outcomes for CP.^18,21,29^

More studies are needed to identify which movement features the deep learning method selects as relevant for CP. A step toward this identification could be localization of movement features within skeleton sequences (eFigure 3 in the Supplement).^40,42^ In the present study, we did not investigate whether the deep learning method used features associated with fidgety movements, other movements, and postural patterns in the early motor repertoire (eg, kicking and body symmetry)^43^ or as yet unidentified patterns of movement.

The comparable performance of the deep learning method vs the observational GMA tool in this study may reflect an upcoming paradigm shift in early prediction of CP. A recent review by Silva et al^18^ highlighted the fact that adoption of automated CP prediction in clinical practice has been restricted because existing machine learning methods lack the predictive accuracy of the GMA. The feasibility of home-based smartphone recordings^22,44,45,46,47^ and associated infant motion tracking^37^ may be combined with the proposed deep learning method to obtain a fully automated system for clinical decision support.

The sensitivity of the observational GMA tool was lower than reported in some previous reviews^13,48^ but similar to findings of other studies.^49,50^ A sensitivity level that was lower than commonly reported^4^ may, at least in part, be explained by the classification of sporadic fidgety movements as normal. This approach contrasted with the classification method taught in courses by the General Movements Trust, but it may increase the accuracy and positive predictive value of the GMA, as reported in a previous study.^31^ Furthermore, a single assessment at approximately 12 weeks’ corrected age may have had a role in the lower sensitivity observed in the present study compared with the sensitivity levels reported by studies performing several assessments throughout the fidgety movement period.^9,51^

The present study included infants recruited from several sites based on a variety of risk factors for perinatal brain injury.^27,31,32,33^ Despite the diverse set of risk factors and clinical characteristics of infants, the prevalence of CP in each diagnostic group matched numbers found in the literature.^52,53,54^ This consistency suggests that the results are generalizable to clinical follow-up programs for infants who were previously in the neonatal intensive care unit based on an increased risk of adverse neurodevelopment.

### Limitations

This study has several limitations. The use of a separate data set for method development limits the number of infants with CP who can be included in the assessment of external validity. This smaller sample limits the possibility of performing subgroup analyses of CP subtypes and GMFCS levels. Further research could assess the validity of the deep learning method on different types of CP and separate medical risk factors. Few children were assessed for CP before age 2 years, which may have resulted in lack of identification of several children with mild phenotypes. Short follow-up duration may also have produced less accurate GMFCS classification because of lower reliability among children younger than 2 years.^55^ However, inaccurate GMFCS classification of a few children is unlikely to change the general interpretation of results because classification rarely changes from ambulatory CP to nonambulatory CP and vice versa.^55^

The present study included videos recorded using a standardized setup; therefore, the deep learning–based CP prediction method requires validation using home-based smartphone recordings. The prediction model may also be refined by extending the skeleton sequence to include facial expressions and fine motor function in fingers and toes that may be associated with CP^43^ and by including techniques, such as temporal attention,^40^ to enable varying influence of the CP risk of different 5-second windows in the skeleton sequence.

## Conclusions

In this prognostic study, the novel deep learning–based CP prediction model had predictive accuracy comparable with GMA results among an external multicenter sample of infants at high risk. The predictive model also differentiated between infants with ambulatory vs nonambulatory CP and infants with spastic unilateral vs spastic bilateral CP. A fully automated movement analysis for CP prediction may serve as an important decision support for clinicians caring for infants at high risk.^18,21^ Future research is needed to identify specific movement biomarkers associated with CP outcome and facilitate widespread clinical use.

## Notes

Supplement. **eAppendix 1.** Related Previously Published Articles **eAppendix 2.** Characteristics of Included Infants **eAppendix 3.** Deep Learning–Based CP Prediction Model **eAppendix 4.** Decision Thresholds **eTable 1.** Characteristics of Infants in Data Sets **eTable 2.** Internal Validation of Deep Learning Method **eTable 3.** Search Space of 20 Architectural Choices **eTable 4.** Characteristics of Architectures Obtained by K-Best Search **eTable 5.** Predictive Values of Decision Thresholds on External Validation **eFigure 1.** Overall Architecture of Graph Convolutional Networks **eFigure 2.** ROC Curves on External Validation **eFigure 3.** Class Activation Mapping
eReferences
