# Supplementary material for: Development and Validation of a Deep Learning Method to Predict Cerebral Palsy From Spontaneous Movements in Infants at High Risk
Source: JAMA Netw Open. 2022 Jul 11;5(7):e2221325. doi: 10.1001/jamanetworkopen.2022.21325 (PMC9274325; doi:10.1001/jamanetworkopen.2022.21325)
Supplement: Supplement. — eAppendix 1. Related Previously Published Articles eAppendix 2. Characteristics of Included Infants eAppendix 3. Deep Learning–Based CP Prediction Model eAppendix 4. Decision Thresholds eTable 1. Characteristics of Infants in Data Sets eTable 2. Internal Validation of Deep Learning Method eTable 3. Search Space of 20 Architectural Choices eTable 4. Characteristics of Architectures Obtained by K-Best Search eTable 5. Predictive Values of Decision Thresholds on External Validation eFigure 1. Overall Architecture of Graph Convolutional Networks eFigure 2. ROC Curves on External Validation eFigure 3. Class Activation Mapping eReferences [file jamanetwopen-e2221325-s001.pdf]

Supplementary Online Content

Groos D, Adde L, Aubert S, et al. Development and validation of a deep learning method to predict cerebral palsy from spontaneous movements in infants at high risk. *JAMA Netw Open*. 2022;5(7):e2221325. doi:10.1001/jamanetworkopen.2022.21325

eAppendix 1. Related Previously Published Articles

eAppendix 2. Characteristics of Included Infants

eAppendix 3. Deep Learning–Based CP Prediction Model

eAppendix 4. Decision Thresholds

eTable 1. Characteristics of Infants in Data Sets

eTable 2. Internal Validation of Deep Learning Method

eTable 3. Search Space of 20 Architectural Choices

eTable 4. Characteristics of Architectures Obtained by K-Best Search

eTable 5. Predictive Values of Decision Thresholds on External Validation

eFigure 1. Overall Architecture of Graph Convolutional Networks

eFigure 2. ROC Curves on External Validation

eFigure 3. Class Activation Mapping

eReferences

This supplementary material has been provided by the authors to give readers additional information about their work.

© 2022 Groos D et al. *JAMA Network Open*.

eAppendix 1. Related Previously Published Articles

The present study is related to five previously published papers:

Støen et al.[1]: This study (Støen et al.[1]) did not assess a Machine Learning-based CP prediction but was a study of GMA and its predictive accuracy for CP. The present study harnessed video recordings, GMA classifications, and CP outcomes of the infant sample from Norway and United States collected by Støen et al.[1]

Adde et al.[2]: This study (Adde et al.[2]) evaluated a simple statistical method for Conventional Machine Learning-based CP prediction without assessing the external validity. The Machine-Learning method used was entirely different from the method presented in the present study. The present study harnessed video recordings, GMA classifications, and CP outcomes of the infant sample from Norway collected by Adde et al.[2]

Pascal et al.[3]: This study (Pascal et al.[3]) did not assess a Machine Learning-based CP prediction but assessed the prediction of CP using GMA. The present study harnessed video recordings, GMA classifications, and CP outcomes of the infant sample from Belgium collected by Pascal et al.[3]

Aker et al.[4]: This study (Aker et al.[4]) did not assess a Machine Learning-based CP prediction but assessed CP prediction using GMA. The present study harnessed video recordings, GMA classifications, and CP outcomes of the infant sample from India collected by Aker et al.[4]

Ihlen et al.[5]: The present study and the study by Ihlen et al.[5] both harnessed the video recordings, GMA classifications, and CP outcomes of the infant sample from Norway and United States collected by Støen et al.[1] but the previous study of Ihlen et al.[5] evaluated a semiautomated Conventional Machine-Learning method for CP prediction, in contrast to the fully automated Deep-Learning method of the present study. The study of Ihlen et al.[5] neither assessed the external validity of the Conventional Machine-Learning method.

© 2022 Groos D et al. *JAMA Network Open*.

eAppendix 2. Characteristics of Included Infants

The study sample comprised infants from four previous studies[1-4]. The first study[1] enrolled infants who were referred to neurodevelopmental follow-up at discharge from tertiary care NICUs at three sites in Norway and two sites in the United States, including a) preterm infants with a gestational age (GA) < 29 weeks and/or birthweight (BW) < 1000 g (n=119), b) infants with congenital heart disease (CHD) in need of cardiac surgery before 4 weeks of age (n=41), c) infants with neonatal arterial ischemic stroke (NAIS; n=9), d) infants with neonatal encephalopathy (NE; n=43), e) infants with other risk factors for adverse neurological development (e.g. congenital anomalies and/or chromosomal abnormalities with extended NICU stay beyond 10 weeks CA, neonatal seizures, central nervous system (CNS) abnormalities, abnormal neonatal imaging, CNS infection, severe hypoglycemia; n=80), and f) infants with GA < 31 weeks and/or BW < 1500 g, enrolled in a randomized controlled trial of two different doses of inhaled nitric oxide for neuroprotection, requiring oxygen at birth (NOVA2 trial; https://clinicaltrials.gov/ct2/show/NCT00515281; n=116). The second study[2] recruited infants from 4 sites in Norway, including a) preterm infants with GA < 28 weeks and/or BW < 1000 g (n=15), b) infants with NE (n=4), c) infants with NAIS (n=2), and d) infants with other risk factors for adverse neurological development (n=9). The third study[3] comprised infants with NAIS from six sites in Belgium (n=37). The fourth study[4] comprised infants admitted to a tertiary care NICU in South India with NE (n=82). See eTable 1 for descriptive statistics, including cerebral palsy (CP) status, of infants in datasets for training & validation (i.e., method development) and test set (i.e., external validation) and study affiliation.

© 2022 Groos D et al. *JAMA Network Open*.

eAppendix 3. Deep Learning–Based CP Prediction Model

In the following section, we describe stepwise how the Deep Learning-based prediction model was developed:

Step 1: The skeleton sequence was resampled to 30 Hz and a 5-point temporal median filter was applied to each skeletal coordinate time series. Subsequently, the skeleton sequence was centralized according to the median mid pelvis location and normalized by two times the trunk length of the infant (i.e., median distance from upper chest to mid pelvis).

Step 2: The skeleton sequence was divided into 5 second windows comprising T $(= 5\ s \cdot 30\ s^{-1} = 150)$ time steps of J $(= 19)$ body keypoints (i.e., joints), each with D $(= 2)$ coordinates (i.e., $x_{t,j}$ and $y_{t,j}$ for keypoint j at time step t). In each 5 second window, the infant skeletons were rotated spatially for vertical alignment of upper chest and mid pelvis in the first time step.

Step 3: Biomechanical properties, i.e., position $\boldsymbol{p}_{t,v}$ and velocity $\boldsymbol{v}_{t,j}$ of each skeletal keypoint and distance from the neighboring body keypoint $\boldsymbol{b}_{t,j} = \boldsymbol{p}_{t,j} - \boldsymbol{p}_{t,j_{adj}}$, were defined for each time step in a 5 second window and used as input variables to the Deep Learning-based CP prediction model.

Step 4: The processing of the input variables was performed by an ensemble of Graph Convolutional Networks (GCNs) (i.e., artificial expert instances) where the overall architecture is illustrated in eFigure 1. The configurations of the input branches, main branch, and pooling layer in eFigure 1 and their general properties were determined by *K*-Best Search[6] with the search space summarized in eTable 3. The performance of GCN architectures was evaluated by the area under the receiver operating characteristic curve (AUC) on internal validation folds of the dataset. All GCNs were optimized using He initialization[7], Stochastic Gradient Descent with learning rate of $5 \cdot 10^{-4}$ and Nesterov momentum of 0.9, and batch size of 32 on an NVIDIA Tesla V100 GPU. Five second windows were randomly sampled from the skeleton sequence and data augmentation with scaling (0.7 – 1.3), rotation (+/- 45 degrees), and translation (+/- 0.3) was employed to avoid overfitting. The best performing GCN architectures from 10 iterations of *K*-Best Search (i.e., artificial experts), as summarized in eTable 4, were trained on 1 377 600 – 1 380 000 and validated on 5 962 – 6 501 5 second windows during 200 epochs for each of the seven folds in the cross-validation, comprising seven sets of model parameters associated with each of the GCN architectures.

Step 5: The seven versions of the 10 obtained GCN models in eTable 4 constituted the 70 artificial expert instances. The artificial expert instances were utilized to yield CP predictions according to eFigure 1 on unseen skeleton sequences (14 697 5 second windows) of the test set with 2.5 seconds overlap between each 5 second window.

© 2022 Groos D et al. *JAMA Network Open*.

eAppendix 4. Decision Thresholds

From the receiver operating characteristic (ROC) curve in eFigure 2, we observe there are several feasible options for the choice of decision threshold in the Deep Learning-based CP prediction, depending on the preference of high sensitivity and few false negatives (e.g., decision threshold of 0.15), high specificity and few false positives (e.g., 0.35), or a compromise of the two (e.g., 0.25), with an overall area under the ROC curve of 0.92. In eTable 5, the distributions of uncertainty of predictions across different decision thresholds are also presented.

© 2022 Groos D et al. *JAMA Network Open*.

eTable 1. Characteristics of Infants in Data Sets

	**Heterogeneous high-risk infants[1,2]**		**Infants with perinatal stroke[3]**		**Infants with neonatal encephalopathy[4]**	
	Training & validation	*Test*	*Training & validation*	*Test*	*Training & validation*	*Test*
No. infants (%)	328 (78.5)	110 (79.1)	28 (6.7)	9 (6.5)	62 (14.8)	20 (14.4)
GA, mean (SD), w.	31.7 (6.2)	30.6 (5.9)	34.8 (5.4)	36.4 (5.1)	39.1 (1.4)	39.4 (1.2)
BW, mean (SD), g	1849 (1245)	1678 (1209)	2430 (1088)	2669 (1209)	2904 (512)	2948 (555)
Sex, no. (%)						
Male	181 (55.2)	57 (51.8)	17 (60.7)	5 (55.6)	39 (62.9)	11 (55.0)
Female	147 (44.8)	53 (48.2)	11 (39.3)	4 (44.4)	23 (37.1)	9 (45.0)
CA rec., mean (SD), w.	12.3 (1.3)	12.2 (1.4)	12.0 (1.6)	11.1 (0.8)	11.2 (1.8)	12.0 (1.7)
CA fol., mean (SD), m.	40.5 (15.7)	39.7 (17.3)	27.9 (5.3)	27.7 (6.2)	19.4 (4.3)	20.8 (6.8)
CP diagnosis, no. (%)	41 (12.5)	14 (12.7)	10 (35.7)	3 (33.3)	12 (19.4)	4 (20.0)
CP subtype, no. (%)						
Spastic unilateral	12 (29.3)	4 (28.6)	8 (80.0)	2 (66.7)	1 (8.3)	1 (25.0)
Spastic bilateral	22 (53.7)	9 (64.3)	2 (20.0)	1 (33.3)	9 (75.0)	3 (75.0)
Dyskinetic	5 (12.2)				1 (8.3)	
Ataxic	1 (2.4)					
Not available	1 (2.4)	1 (7.1)			1 (8.3)	
GMFCS, no. (%)						
I	13 (31.7)	2 (14.3)	3 (30.0)	2 (66.7)	2 (16.7)	2 (50.0)
II	5 (12.2)		4 (40.0)		1 (8.3)	1 (25.0)
III	3 (7.3)	5 (35.7)	1 (10.0)		1 (8.3)	
IV	8 (19.5)	3 (21.4)	1 (10.0)	1 (33.3)	2 (16.7)	
V	11 (26.8)	3 (21.4)			5 (41.7)	1 (25.0)
Not available	1 (2.4)	1 (7.1)	1 (10.0)		1 (8.3)	

Abbreviations: GA, gestational age; w., weeks; BW, birth weight; CA, corrected age; rec., recording; fol., follow-up; m., months; CP, cerebral palsy; GMFCS, Gross Motor Function Classification System.

© 2022 Groos D et al. *JAMA Network Open*.

eTable 2. Internal Validation of Deep Learning Method

TP	FP	TN	FN	Sensitivity %	Specificity %	PPV %	NPV %	Accuracy %
45	16	339	18	71.4 [58.7, 82.1]	95.5 [92.8, 97.4]	73.8 [60.9, 84.2]	95.0 [92.2, 97.0]	91.9 [88.8, 94.3]

The internal validity was evaluated using 7-fold cross-validation, with sensitivity fixed at the level of GMA (i.e., 70.0%). All values are provided in percentages, along with 95% confidence interval.
Abbreviations: TP, true positives; FP, false positives; TN, true negatives; FN, false negatives; PPV, positive predictive value; NPV, negative predictive value.

© 2022 Groos D et al. *JAMA Network Open*.

eTable 3. Search Space of 20 Architectural Choices

	No.	Architectural choice	Alternatives
Input branches	1	No. modules of input branches	1, 2, 3
	2	Width of input branches	6, 8, 10, 12
	3	Block type in initial module[a]	Basic[8], Bottleneck[8], MBConv[9]
	4	Residual type in initial module[a]	None, Block[8], Module[8], Dense[8]
	5	No. temporal scales in input branches	1, 2, 3, Linear[b]
Main branch	6	No. levels of main branch	1, 2
	7	No. modules of main branch levels	1, 2, 3
	8	Width of first level of main branch[c]	6, 8, 10, 12
	9	No. temporal scales in main branch	1, 2, 3, Linear[b]
Pooling layer	10	Pooling layer type	Global average, Spatial average
General properties	11	Graph convolution type	Spatial configuration[10], DA 2[11,d], DA 4[11,d], DA 4+2[11,d]
	12	Block type[e]	Basic[8], Bottleneck[8], MBConv[9]
	13	Bottleneck factor	2, 4
	14	Residual type[e]	None, Block[8], Module[8], Dense[8]
	15	SE type	None, Inner[12], Outer[12], Both[12]
	16	SE ratio	2, 4
	17	SE ratio type	Relative, Absolute
	18	Attention type	None, Channel[8], Frame[8], Joint[8]
	19	Nonlinearity type	ReLU[13], Swish[13]
	20	Temporal kernel size	3, 5, 7, 9

Abbreviations: MBConv, mobile inverted bottleneck convolution; DA, disentangled aggregation; SE, Squeeze-and-Excitation; ReLU, rectified linear unit.
[a] The initial module is the first module of input branches.
[b] Linear scaling indicates that number of temporal scales increases by one for each module.
[c] For the second level of the main branch, the width is doubled, while also reducing the time dimension by a factor of 2.
[d] Graph convolutions with disentangled aggregation have different number of hops in neighborhood (i.e., 2 or 4), where 4+2 yields separate number of hops in input modules and main module, with 4 and 2, respectively.
[e] There is a separate architecture choice associated with the initial module.

© 2022 Groos D et al. *JAMA Network Open*.

eTable 4. Characteristics of Architectures Obtained by K-Best Search

	***K*-Best Search**									
**Architectural choice**	**1**	**2**	**3**	**4**	**5**	**6**	**7**	**8**	**9**	**10**
No. modules of input br.	3	2	3	3	2	3	1	2	3	2
Width of input br.	10	10	12	10	10	12	8	6	6	12
Block type in initial mod.	Bottl.	Basic	Basic	Basic	Bottl.	Basic	Basic	MBC.	Bottl.	Basic
Residual type in initial mod.	None	Den.	None	Block	Den.	Den.	Mod.	Block	Den.	Den.
No. tmp. scales in input br.	1	3	2	2	3	1	3	2	1	2
No. levels of main br.	1	1	1	1	2	2	2	2	2	1
No. modules of main br. levels	1	3	2	1	1	3	3	2	1	3
Width of first level of main br.	12	12	8	8	6	10	12	12	12	10
No. tmp. scales in main br.	1	2	2	Lin.	3	Lin.	3	1	Lin.	3
Pooling layer type	Gl.	Gl.	Gl.	Sp.	Gl.	Gl.	Sp.	Gl.	Sp.	Sp.
Graph convolution type	DA 2	DA 4+2	SC	DA 4	DA 4	DA 2	DA 2	DA 4	DA 2	SC
Block type	Basic	MBC.	Basic	Basic	Basic	Bottl.	Basic	Basic	Basic	Basic
Bottl. factor	4	2	2	4	2	4	4	4	4	4
Residual type	None	Block	Mod.	Den.	None	Block	None	Den.	None	None
SE type	None	Outer	Inner	None	Outer	None	None	Outer	Outer	None
SE ratio	-	4	2	-	2	-	-	4	4	-
SE ratio type	-	Abs.	Abs.	-	Abs.	-	-	Abs.	Abs.	-
Attention type	Ch.	Ch.	None	Ch.	Ch.	Ch.	None	None	Ch.	Ch.
Nonlinearity type	ReLU	Sw.	ReLU	Sw.	ReLU	Sw.	Sw.	ReLU	ReLU	Sw.
Tmp. kernel size	9	7	7	7	7	3	9	5	9	7
AUC on external validation	0.903	0.903	0.889	0.893	0.902	0.910	0.905	0.889	0.917	0.893

AUC on external validation of each model architecture was evaluated as the mean AUC across the seven sets of model parameters from 7-fold cross-validation.
Abbreviations: br., branch; bottl., bottleneck; MBC., mobile inverted bottleneck convolution; den., dense; mod., module; tmp., temporal; lin., linear; gl., global; sp., spatial; DA, disentangled aggregation; SC, spatial configuration; SE, Squeeze-and-Excitation; abs., absolute; ch., channel; ReLU, rectified linear unit; sw., swish; AUC, area under the receiver operating characteristic curve.

© 2022 Groos D et al. *JAMA Network Open.*

eTable 5. Predictive Values of Decision Thresholds on External Validation

Thres.	TP	FP	TN	FN	Sens. %	Spec. %	PPV %	NPV %	Acc. %	Infants with CP				Infants without CP			
0.15	20	30	88	1	95.2	74.6	40.0	98.9	77.7	17	3	0	1	70	18	18	12
0.20	18	20	98	3	85.7	83.1	47.4	97.0	83.5	14	4	2	1	78	20	10	10
0.25	18	15	103	3	85.7	87.3	54.6	97.2	87.1	13	5	2	1	87	16	9	6
0.30	16	14	104	5	76.2	88.1	53.3	95.4	86.3	12	4	4	1	95	9	10	4
0.35	15	7	111	6	71.4	94.1	68.2	94.9	90.6	12	3	4	2	102	9	5	2

Red and orange color coding represent certain and uncertain classification into CP, whereas green and yellow represent certain and uncertain classification into no CP.
Abbreviations: thres., decision threshold; TP, true positives; FP, false positives; TN, true negatives; FN, false negatives; sens., sensitivity; spec., specificity; PPV, positive predictive value; NPV, negative predictive value; acc., accuracy.

© 2022 Groos D et al. *JAMA Network Open*.

eFigure 1. Overall Architecture of Graph Convolutional Networks

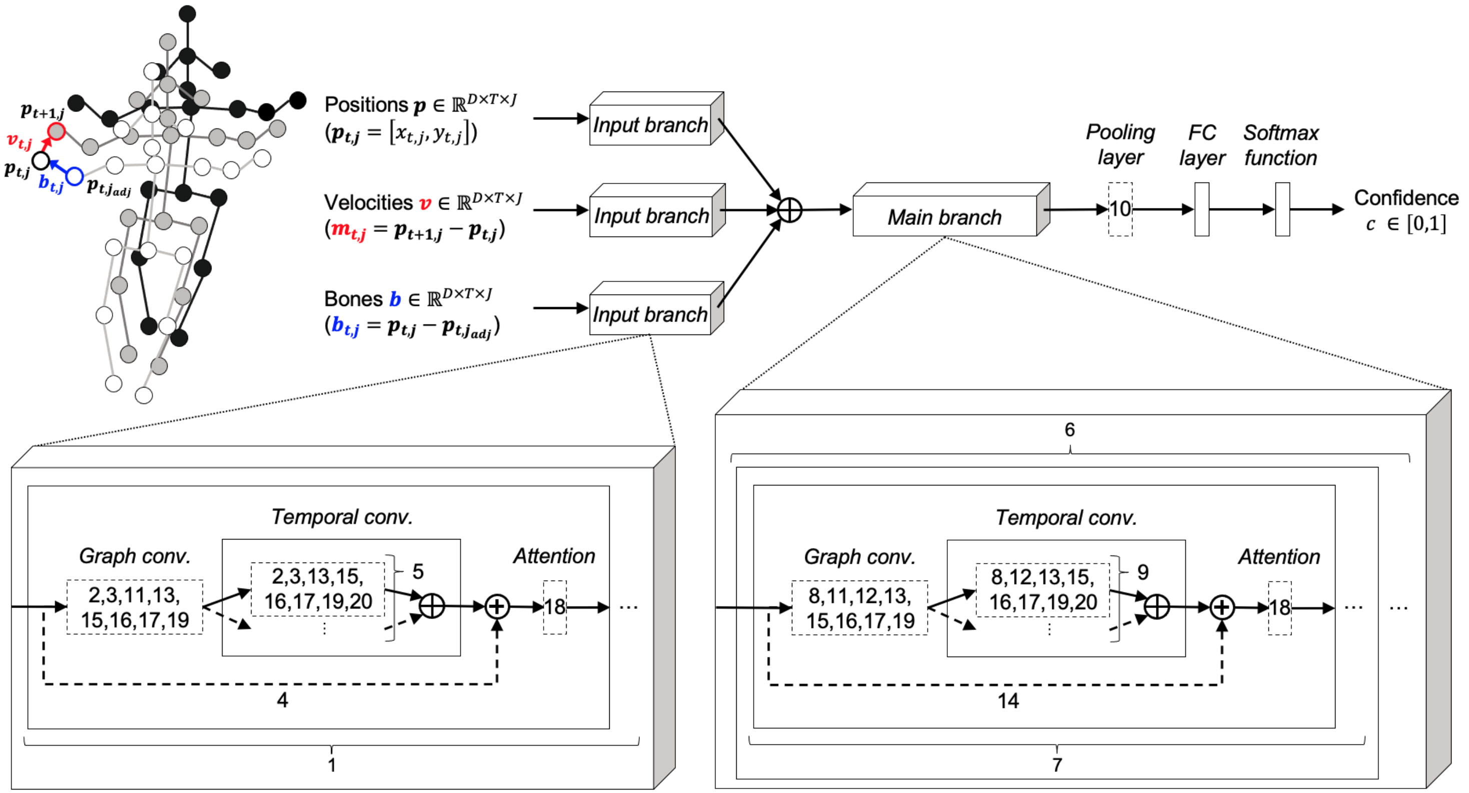

From left: A GCN processes biomechanical properties, describing positions p, velocities (change in position) v, and bones (distance from the neighboring body keypoint) b, of a window of T time steps from a skeleton sequence, J body keypoints (joints), and D spatial dimensions, through parallel input branches, followed by a main branch, pooling layer, fully connected (FC) layer, and softmax function to yield confidence c about the risk of CP from 0.0 (no CP) to 1.0 (CP). Architectural choices are labeled according to the numbering in eTable 3.

© 2022 Groos D et al. *JAMA Network Open*.

eFigure 2. ROC Curves on External Validation

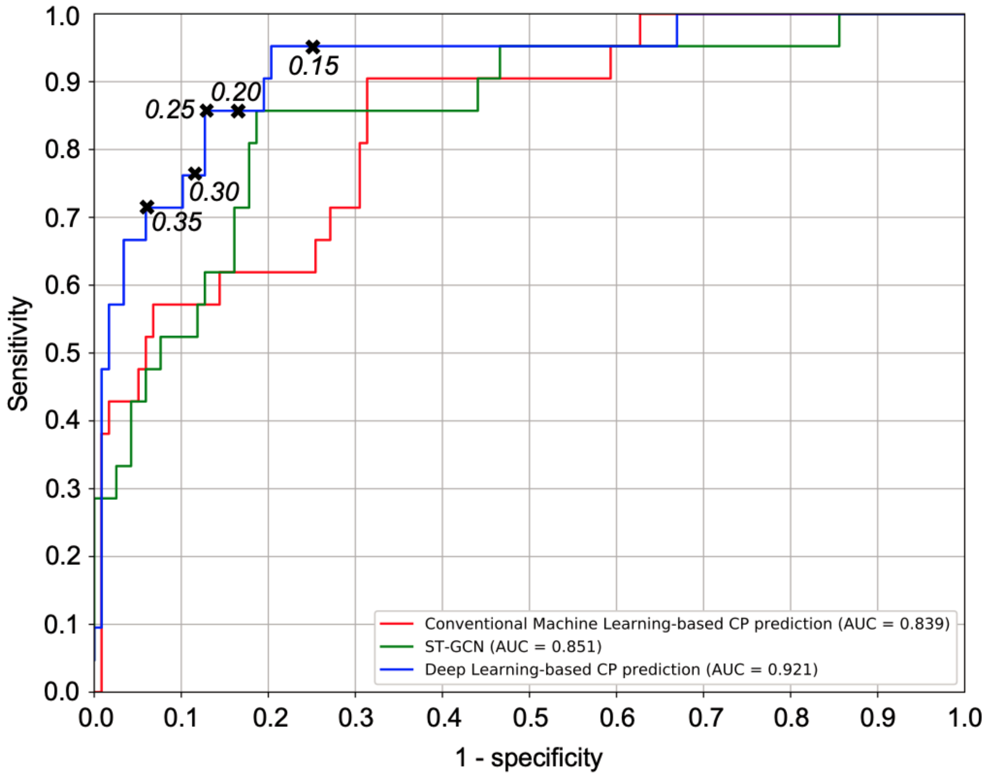

Receiver operating characteristic (ROC) curve and area under the ROC curve (AUC) of the Deep Learning-based CP prediction in relation to the Conventional Machine Learning-based CP prediction[5] and ST-GCN[10]. Different decision thresholds (0.15, 0.20, 0.25, 0.30, and 0.35) of the Deep-Learning method are marked with cross and text in italic.

© 2022 Groos D et al. *JAMA Network Open*.

eFigure 3. Class Activation Mapping

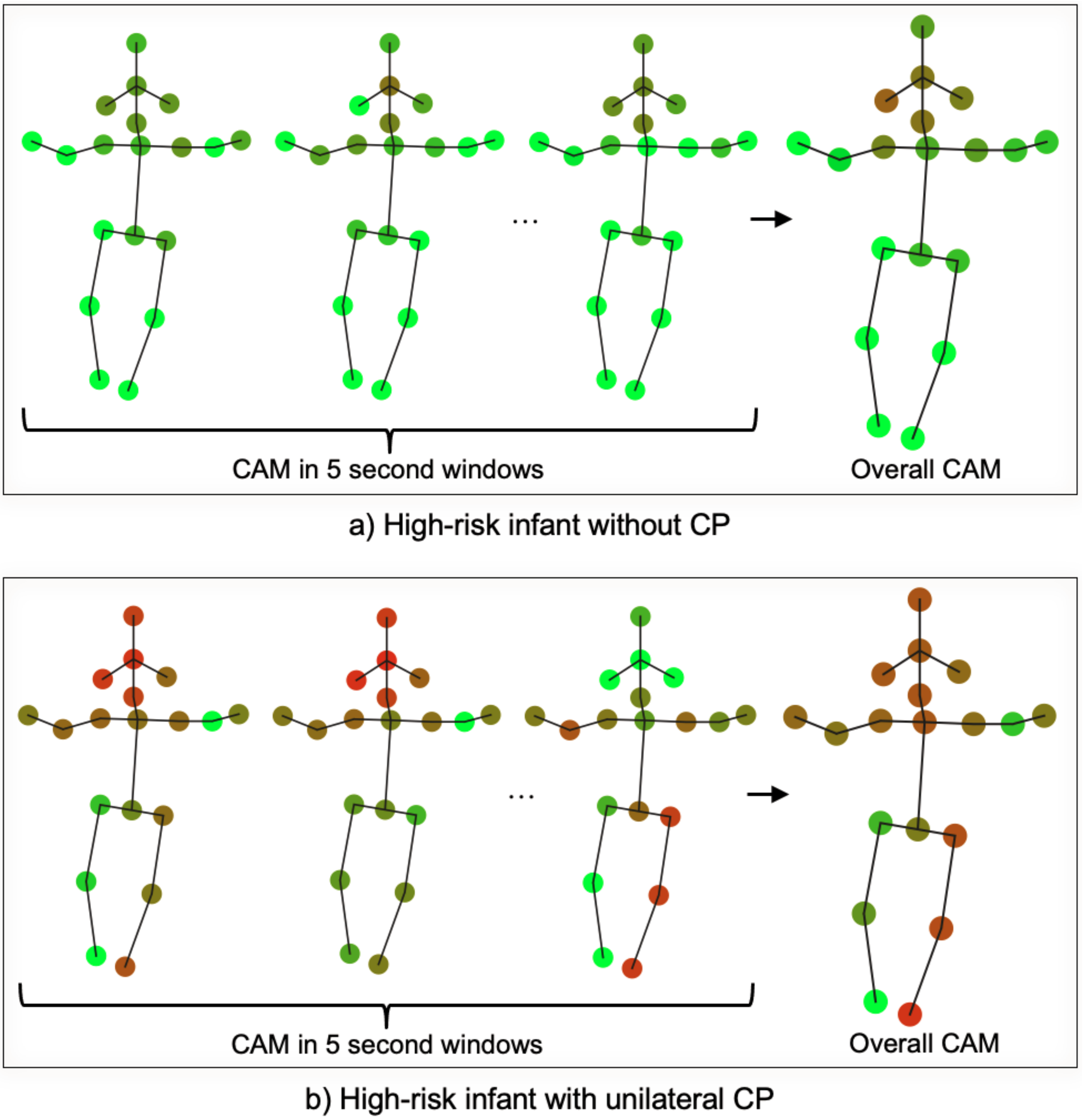

Class activation mapping[14,15] (CAM) visualizes the contribution of each body keypoint towards the CP risk in individual 5 second windows, where keypoints with no or minimal contribution towards CP risk are colored green, whereas red body keypoints have high contribution towards CP risk. An overall CAM of a skeleton sequence can be computed as the median contribution of each body keypoint across all 5 second windows, as indicated for a) one representative high-risk infant without CP and b) one representative high-risk infant with unilateral CP. Accordingly, CAM facilitates spatial localization of movements related to CP and no CP.

© 2022 Groos D et al. *JAMA Network Open*.

eReferences

1. Støen R, Boswell L, De Regnier RA, et al. The predictive accuracy of the general movement assessment for cerebral palsy: a prospective, observational study of high-risk infants in a clinical follow-up setting. *J Clin Med.* 2019;8(11):1790. doi:10.3390/jcm8111790.

2. Adde L, Helbostad JL, Jensenius AR, Taraldsen G, Grunewaldt KH, Støen R. Early prediction of cerebral palsy by computer-based video analysis of general movements: a feasibility study. *Dev Med Child Neurol.* 2010;52(8):773-8. doi:10.1111/j.1469-8749.2010.03629.x.

3. Pascal A, Govaert P, Ortibus E, et al. Motor outcome after perinatal stroke and early prediction of unilateral spastic cerebral palsy. *Eur J Paediatr Neurol.* 2020;29:54-61. doi:10.1016/j.ejpn.2020.09.002.

4. Aker K, Thomas N, Adde L, et al. Prediction of outcome from MRI and general movements assessment after hypoxic-ischaemic encephalopathy in low-income and middle-income countries: data from a randomised controlled trial. *Arch Dis Child Fetal Neonatal Ed.* 2022;107(1):32-38. doi:10.1136/archdischild-2020-321309.

5. Ihlen EA, Støen R, Boswell L, et al. Machine learning of infant spontaneous movements for the early prediction of cerebral palsy: A multi-site cohort study. *J Clin Med.* 2019;9(1):5. doi:10.3390/jcm9010005.

6. Groos D. Convolutional networks for video-based infant movement analysis: Towards objective prognosis of cerebral palsy from infant spontaneous movements. *NTNU.* 2022 (submitted thesis).

7. He K, Zhang X, Ren S, Sun J. Delving deep into rectifiers: Surpassing human-level performance on imagenet classification. *Proceedings of the IEEE international conference on computer vision.* 2015;1026-1034. doi:10.1109/ICCV.2015.123.

8. Song YF, Zhang Z, Shan C, Wang L. Stronger, faster and more explainable: A graph convolutional baseline for skeleton-based action recognition. *Proceedings of the 28th ACM International Conference on Multimedia.* 2020;1625-1633. doi:10.1145/3394171.3413802.

9. Tan M, Le Q. EfficientNet: Rethinking model scaling for convolutional neural networks. *Proceedings of the 36th International Conference on Machine Learning, PMLR.* 2019;97:6105-6114.

10. Yan S, Xiong Y, Lin, D. Spatial temporal graph convolutional networks for skeleton-based action recognition. *Thirty-second AAAI conference on artificial intelligence*. 2018.

11. Liu Z, Zhang H, Chen Z, Wang Z, Ouyang W. Disentangling and unifying graph convolutions for skeleton-based action recognition. *Proceedings of the IEEE/CVF conference on computer vision and pattern recognition.* 2020;143-152. doi:10.1109/CVPR42600.2020.00022.

12. Hu J, Shen L, Sun G. Squeeze-and-excitation networks. *Proceedings of the IEEE conference on computer vision and pattern recognition.* 2018;7132-7141. doi:10.1109/CVPR.2018.00745.

13. Ramachandran P, Zoph B, Le QV. Searching for activation functions. *arXiv.* 2017;1710.05941.

14. Zhou B, Khosla A, Lapedriza A, Oliva A, Torralba A. Learning deep features for discriminative localization. *Proceedings of the IEEE conference on computer vision and pattern recognition*. 2016;2921-2929.

15. Song YF, Zhang Z, Shan C, Wang L. Constructing stronger and faster baselines for skeleton-based action recognition. *IEEE Transactions on Pattern Analysis and Machine Intelligence.* 2022. doi:10.1109/TPAMI.2022.3157033.

© 2022 Groos D et al. *JAMA Network Open.*
